# Utilizing Implementation Science to Accelerate Adoption of a Program to Prevent Avoidable Transfers

**DOI:** 10.1093/geroni/igaa057.2745

**Published:** 2020-12-16

**Authors:** Karen Southard, Suzie Kaye

**Affiliations:** 1 The Carolinas Center for Medical Excellence (CCME), Columbia, South Carolina, United States; 2 Florida Atlantic University, Boca Raton, Florida, United States

## Abstract

The research team combined their long-term care management experience with feedback from the 16 pilot nursing homes and several hundred workshop and webinar participants’ reports to identify the most effective strategies for implementation of the Guide and smaller Trifold. The learning framework employed to develop these Best Practices was directed to two distinct audiences 1) the licensed staff of the facility and 2) the residents of the facilities and their families. Critical elements identified included: when and how to distribute the Guide; how the Guide can be used to support difficult conversations e.g. about end of life choices; and effective and efficient ways to train staff. The framework for discussion during the symposium will be achievement of organizational readiness and the relevance of the four E’s of implementation science as it applies to long term care organizations.

